# Association of Maternal Folate Intake and Offspring MTHFD1 and MTHFD2 Genes with Congenital Heart Disease

**DOI:** 10.3390/nu15163502

**Published:** 2023-08-09

**Authors:** Hanjun Liu, Jun Ou, Yige Chen, Qian Chen, Manjun Luo, Tingting Wang, Jiabi Qin

**Affiliations:** 1 Department of Epidemiology and Health Statistics, Xiangya School of Public Health, Central South University, Changsha 410078, China; 226911022@csu.edu.cn (H.L.); 226901013@csu.edu.cn (J.O.); 226911018@csu.edu.cn (Y.C.); 216912048@csu.edu.cn (Q.C.); luomj0810@csu.edu.cn (M.L.); 2 National Health Committee Key Laboratory of Birth Defect for Research and Prevention, Hunan Provincial Maternal and Child Health Care Hospital, Changsha 410028, China

**Keywords:** congenital heart defect, folic acid supplementation, methylenetetrahydrofolate dehydrogenase gene, interaction

## Abstract

Existing evidence supported that congenital heart defect (CHD) was associated with a combination of environmental and genetic factors. Based on this, this study aimed at assessing the association of maternal folic acid supplementation (FAS), genetic variations in offspring methylenetetrahydrofolate dehydrogenase (MTHFD)1 and MTHFD2 genes, and their interactions with CHD and its subtypes. A hospital-based case–control study, including 620 cases with CHD and 620 healthy children, was conducted. This study showed that the absence of FAS was significantly associated with an increased risk of total CHD and its subtypes, such as atrial septal defect (ASD). FAS during the first and second trimesters was associated with a significantly higher risk of CHD in offspring compared to FAS during the three months prior to conception. The polymorphisms of offspring MTHFD1 and MTHFD2 genes at rs2236222, rs11849530, and rs828858 were significantly associated with the risk of CHD. Additionally, a significantly positive interaction between maternal FAS and genetic variation at rs828858 was observed for the risk of CHD. These findings suggested that pregnant women should carefully consider the timing of FAS, and individuals with higher genetic risk may benefit from targeted folic acid supplementation as a preventive measure against CHD.

## 1. Introduction

Congenital heart defect (CHD) is the most prevalent congenital malformation worldwide and has emerged as the leading cause of death among children under five years old in China [1,2]. While surgical interventions have improved survival rates, CHD patients often experience long-term cardiovascular and systemic complications, leading to substantial economic burdens [3,4]. However, the underlying causes and mechanisms of CHD are complex and not fully elucidated. Available evidence indicated that both genetic and environmental factors contribute to the development of CHD [5]. Consequently, investigating the etiology of CHD holds immense social and economic importance.

Cell division and tissue formation are crucial processes during embryonic development. Folic acid, an essential component for DNA synthesis and repair, plays a vital role in these processes. Thus, ensuring an adequate supply of folic acid is imperative for normal embryonic development [6,7]. A study conducted in Beijing, China, demonstrated the effectiveness of maternal folic acid supplementation (FAS) in preventing CHD in offspring, particularly critical cases [8]. However, research conducted in Canada and Norway yielded contrasting results, showing no association between maternal FAS and offspring CHD, even indicating an increased risk of septal defects [9,10]. These discrepancies could be attributed to several factors: (1) variations in the timing of maternal FAS, with some mothers initiating folic acid intake late in pregnancy; (2) inconsistencies in the specific CHD subtypes investigated and the absence of standardized diagnostic criteria across different studies; and (3) the possibility that maternal FAS may only prevent certain CHD subtypes while lacking an effect on others. In an effort to safeguard the well-being of expectant mothers and fetuses in China, a folic acid policy has been implemented. This policy allows pregnant women to receive folic acid supplements prior to conception, during pregnancy, and after delivery. The specific dosage of folic acid is determined based on the individual health and pregnancy conditions of each woman [11]. Although this policy has led to an increase in folic acid usage among pregnant women, its compliance was still influenced by various factors. Research indicated that socioeconomic aspects, such as educational attainment and family financial status, played a role in determining folic acid intake among pregnant women [12,13]. Another significant contributing factor to the low compliance rates was the inadequate understanding of folic acid among expectant mothers in China [14,15]. To address these issues, this study aimed to categorize the timing of maternal FAS and CHD subtypes more comprehensively, in order to explore the association between maternal FAS and CHD in a more comprehensive manner and minimize existing limitations.

The methylene tetrahydrofolate dehydrogenase 1 (MTHFD1) and MTHFD2 genes, despite their importance in single-carbon folate metabolism, received less attention. The MTHFD1 gene encodes a trifunctional enzyme that catalyze the interconversion of single-carbon tetrahydrofolate derivatives. The MTHFD2 gene encodes a nucleo-coded mitochondrial bifunctional enzyme with methylene tetrahydrofolate dehydrogenase and methyltetrahydrofolate cyclohydrolase activities [16]. Disruptions in the function of MTHFD1 and MTHFD2 genes, caused by mutations or abnormalities, can interfere with folic acid metabolism and have adverse effects on cellular biological processes [17]. Previous studies suggested that genetic variations in MTHFD1 and MTHFD2 genes may contribute to the occurrence and development of CHD [18,19,20]. Notably, a study using a mouse model observed that deficiency in MTHFD1 synthetase was associated with a higher incidence of CHD, particularly ventricular septal defects (VSD). This deficiency impaired myocardial growth by inhibiting purine synthesis in rapidly dividing embryonic tissue and impacting DNA replication, thereby limiting cell proliferation [21]. Based on these findings, it is reasonable to believe that MTHFD1 and MTHFD2 genes can influence the occurrence and progression of CHD. However, the current study only focused on the relationship between certain loci of MTHFD1 and MTHFD2 genes (G1958A/rs2236225, rs1950902, etc.) and CHD [18,22,23,24]. Therefore, this study aimed to comprehensively analyze the loci of MTHFD1 and MTHFD2 genes and further investigate the interaction between maternal FAS and MTHFD1 and MTHFD2 genes in relation to CHD. The outcomes of this study will provide a theoretical foundation for more efficient screening of high-risk groups and the development of more effective interventions in the future.

Building upon the aforementioned background, this study aimed to focus on several key aspects. Firstly, it aimed to analyze the association between maternal FAS and CHD, considering both the overall occurrence and specific subtypes of CHD. Additionally, the study sought to investigate the relationship between the timing of folic acid intake and its effectiveness in preventing CHD. Secondly, the research assessed the connection between variations in the MTHFD1 and MTHFD2 genes of offspring and the occurrence of CHD, encompassing various subtypes of the condition. Lastly, the study aimed to explore the potential impact of interactions between maternal FAS and offspring MTHFD1 and MTHFD2 genes on the incidence of CHD.

## 2. Subjects and Methods

### 2.1. Recruitment of Study Participants

The hospital-based case–control study was conducted at Hunan Children’s Hospital from November 2017 to March 2020. The case group included hospitalized patients diagnosed with simple CHD, excluding other congenital diseases, according to the Pediatric and Congenital Cardiac Code (IPCCC). The diagnosis was further confirmed through color Doppler ultrasound or surgical procedures. The control group was randomly chosen from the children’s health care department at Hunan Children’s Hospital during the same time frame. This department primarily emphasizes children’s growth and development and conducts comprehensive health care and examinations for children. To ensure comparability, individuals with CHD and/or other congenital diseases were excluded from the control group after comprehensive medical history inquiries and clinical diagnoses. To mitigate potential recall bias related to maternal exposures during the pre-pregnancy and early pregnancy periods, the age limit for both the case and control groups was set below one year. Moreover, efforts were made to minimize confounding factors stemming from genetic and cultural variances by exclusively enrolling subjects from the Han ethnic group, without any familial relationships between cases and controls. Participants unable to provide samples or unwilling to cooperate in completing the questionnaire were excluded from the study. Sample size estimation followed the formula designed for non-matched case–control studies. The relevant literatures were consulted to identify gene loci (MTHFD1 and MTHFD2) exhibiting statistically significant differences in mutations between the case and control groups [23,25]. Mutation rates for each gene locus in both groups were calculated using data from the literatures. To account for a 20% potential loss of follow-up, a minimum of 530 samples per group (case and control) were deemed necessary. Further information can be found in Table S1. Informed consent forms were obtained from both the case group and the control group, ensuring voluntary participation and a thorough understanding of the research’s purpose and procedures. This study was in accordance with the Declaration of Helsinki, approved by the Ethics Committee of Xiangya School of Public Health, Central South University (approval number: XYGW-2018-07). In addition to this, we have registered this study in the Chinese Clinical Trial Registry Center (registration number: ChiCTR1800016635).

### 2.2. Information and Blood Sample Collection

The study aimed to investigate multiple outcomes, including congenital heart defect (CHD) and its specific subtypes, such as atrial septal defect (ASD), VSD, and patent ductus arteriosus (PDA). A significant variable of interest was maternal FAS. FAS was operationally defined in alignment with the World Health Organization (WHO) guidelines, denoting the consumption of more than 0.4 mg of folic acid daily for a minimum of five days per week, spanning the three months preceding and succeeding pregnancy. Maternal FAS timing was delineated into three distinct intervals: three months prior to conception, the first trimester of pregnancy, and the second trimester of pregnancy.

In this study, professionally trained investigators conducted face-to-face interviews to collect data. Based on our previous studies [18,26], the following covariables were used as confounding factors for subsequent analysis: socio-demographic characteristics (residence, maternal age of pregnancy, pre-pregnancy body mass index (BMI), and child sex), adverse pregnancy history (spontaneous abortion, stillbirth, premature delivery, and low birth weight (LBW)), pre-pregnancy chronic diseases (diabetes), history of pregnancy complications (gestational diabetes and gestational hypertension), and maternal perinatal lifestyle (antibiotic use, perinatal cold, fever, smoking, exposure to second-hand smoke, and drinking). In China, every pregnant woman possesses a “Perinatal Health Handbook” (PHCH), which contains her pregnancy and personal information. After completing the questionnaire collection, additional verification measures were implemented to ensure data accuracy.

Following subject enrollment, 5 mL of peripheral venous blood was collected using an EDTA anticoagulant tube by trained nurses in the department. The collected blood samples were promptly centrifuged at 3500 r/min for 15 min using a low-speed centrifuge. Subsequently, the separated plasma and blood cells were carefully packaged, labeled, and stored in a cryogenic refrigerator at −80 °C. In this study, blood cells were used for SNP detection due to their high sensitivity and reliability.

### 2.3. SNP Selection and Genotyping 

Following established research methods, this study employed a screening process to identify the loci of interest for MTHFD1 and MTHFD2 genes [25]. The procedure involved searching the NCBI database (https://www.ncbi.nlm.nih.gov/SNP/ (accessed on 1 January 2023)) for major MTHFD1 and MTHFD2 genes loci and cross-referencing them with the HapMap database for verification. Loci with an r^2^ value less than 0.8 and a minimum allele frequency (MAF) below 10% were excluded. The detection of single nucleotide polymorphisms (SNPs) followed strict genotyping procedures using the matrix-assisted laser desorption and ionization time-of-flight mass spectrometry MassARRAY system (Agena iPLEX assay, San Diego, CA, USA). Blind tests were conducted on 5–10% of the total samples to assess the reliability of SNP typing results. Furthermore, the experimenter remained unaware of the sample’s origin from either the case or control group to ensure the experiment’s integrity. The success rate of SNP typing exceeded 90%. In this study, 13 genetic loci (rs1950902, rs2236225, rs2236222, rs11849530, rs1256146, rs2236224, rs1256142, rs34616731, rs7571842, rs702466, rs828858, rs828903, and rs1095966) were selected, and their characteristics are presented in Table S2.

### 2.4. Statistical Analysis

The control group underwent a Hardy–Weinberg equilibrium (HWE) test to assess gene frequency balance. To account for multiple comparisons, the *p*-value from the HWE test was adjusted using false discovery rate (FDR) correction to effectively control the false positive rate. Loci with an adjusted *p*-value (Q_FDR_) < 0.1 indicated imbalanced gene frequency and were subsequently eliminated. Three common genetic models were utilized: dominant model (AA vs. Aa + aa), recessive model (AA vs. Aa + aa), and additive model (AA vs. Aa vs. aa). Univariate/multivariate logistic regression analysis was employed to investigate the associations between maternal FAS, various loci, and CHD and its subtypes. The multiplicative interaction effects of maternal FAS with MTHFD1 and MTHFD2 genes on CHD were also examined, along with stratified analyses. In the multivariate logistic regression model, we assessed the significant impact of maternal FAS and MTHFD1 and MTHFD2 gene polymorphisms using adjusted odds ratios (aOR). To control for potential confounding factors, we considered statistically significant variables from the baseline data. Additive interaction was assessed using RERI (Relative Excess Risk due to Interaction) and AP (Attributable Proportion). An additive interaction is absent when the confidence interval (CI) for RERI and AP included 0. A positive RERI and AP, with the CI excluding 0, indicated synergistic interaction. Conversely, a negative RERI and AP, with the CI excluding 0, suggested antagonistic interaction. FAS was analyzed as an exposure factor in the relevant calculations.

In this study, we utilized Epidata 3.1 to establish a database, with two individuals performing simultaneous data entry and reviewing to ensure accuracy. Counting data were presented as case numbers or constituent ratios. Binary variable testing was conducted using either χ^2^ or Fisher’s exact probability test. Wilcoxon test was employed for ordinal multi-categorical variables. Statistical analysis was performed using SPSS25.0 and R (version 4.2.1). A significance level of α = 0.05 and a *p*-value < 0.05 were considered statistically significant. It is important to note that only the risk of total CHD was evaluated in assessing the interactions between maternal FAS and each MTHFD1 and MTHFD2 genes’ locus due to the limited sample size, and the risks of specific subtypes were not analyzed.

## 3. Result

### 3.1. Comparison of Baseline Characteristics across Groups

The flow-chart of the study sample formation is presented in Figure S1. Following the established inclusion and exclusion criteria, a total of 1240 subjects were included in this study, evenly distributed between the case group and the control group, with 620 subjects in each group. The most prevalent subtypes of CHD diagnosed in the case group were ventricular septal defect (VSD: 448 cases, 72.3%), patent ductus arteriosus (PDA: 168 cases, 27.1%), and atrial septal defect (ASD: 139 cases, 22.4%). It should be noted that children may have multiple CHD subtypes, hence the cumulative percentage exceeding 100%. The baseline characteristics of both groups are presented in Table 1, and significant statistical differences (*p* < 0.05) were observed between the case and control groups in the following variables: residence, education level (years), pre-pregnancy BMI, history of stillbirth, history of preterm birth, LBW history, diabetes, history of gestational diabetes, history of gestational hypertension, antibiotic use during perinatal pregnancy, perinatal cold, perinatal fever, pregnancy smoking, exposure to secondhand smoke during perinatal period, and perinatal drinking. 

### 3.2. Association of Maternal Folic Acid Intake with CHD in Offspring

Table S3 presented the exact count and percentage of individuals who received FAS in the CHD and its subtypes’ groups, as well as the control group. It also categorized maternal FAS into three phases based on the timing of supplementation initiation. The control group had a higher proportion of maternal FAS compared to the case group (93.1% vs. 84.8%). Table 2 shows that the absence of FAS was associated with an increased risk of total CHD and ASD in both univariate and multivariate analyses. (CHD: aOR: 0.51, 95%CI: 0.34–0.77; ASD: aOR: 0.33, 95%CI: 0.19–0.58). However, no statistically significant association was observed in the VSD and PDA groups.

Notably, in the total CHD group, FAS during the first (aOR: 1.80, 95%CI: 1.33, 2.43) and second (aOR: 10.43, 95%CI: 3.83, 28.35) trimesters was associated with a significantly higher risk of CHD in offspring compared to FAS during the three months prior to conception. This finding was consistently observed in the various subtypes of CHD (Table 3).

### 3.3. Association of MTHFD1 and MTHFD2 Genes with CHD and Its Subtypes

Following FDR adjustment, the HWE test was performed on 13 loci from two genes in the control group. The results indicated that all the remaining 11 loci in the study passed the HWE test, except for rs1256146 and rs34616731 of the MTHFD1 gene. This suggested that the samples included in this study exhibited good population representation (Table S4).

Table 4 presents the association analysis between SNPs at 11 loci on MTHFD1 and MTHFD2 genes and CHD and its subtypes. Children carrying the genotypes GA and GG at rs2236222 exhibited a heightened risk for AA (GA vs. AA: aOR: 1.56, 95%CI: 1.21–2.02; GG vs. AA: aOR: 2.28, 95%CI: 1.27–4.04), whereas GG at rs11849530 was linked to a reduced risk for AA (GG vs. AA: aOR: 0.46, 95%CI: 0.30–0.71), and TA at rs828858 was associated with a decreased risk when compared to TT (TA vs. TT: aOR: 0.56, 95%CI: 0.41–0.76). Specifically, the dominant model of rs2236222 (aOR: 1.70, 95%CI: 1.34–2.18) and rs828858 (aOR: 0.59, 95%CI: 0.44–0.79), as well as the recessive model of rs11849530 (aOR: 0.55, 95%CI: 0.37–0.77), exhibited significant associations. Additionally, the additive models of these three genes also showed statistical significance (rs2236222: aOR: 1.54, 95%CI: 1.26–1.89; rs11849530: aOR: 0.75, 95%CI: 0.63–0.91; rs828858: aOR: 0.61, 95%CI: 0.45–0.79). Based on these findings, subsequent interaction analyses with maternal FAS were performed specifically focusing on these three loci.

In the analysis of the three subtypes, the ASD and VSD groups exhibited results consistent with the CHD group. Specifically, the genotype distribution of rs2236222, rs11849530, and rs828858 showed statistical significance, and the additive models of these three loci also demonstrated statistical significance. However, in the PDA group, no locus showed a statistically significant genotype distribution.

### 3.4. Interaction between Maternal Folic Acid Intake and Offspring MTHFD1 and MTHFD2 Genes

Table 5 presents the impact of the multiplicative interaction between maternal FAS and offspring MTHFD1 and MTHFD2 genes on the risk of CHD. The multiplicative interaction with offspring rs828858 showed statistical significance in relation to CHD risk (aOR: 0.26, 95CI%: 0.10, 0.65, *p* = 0.004). However, no multiplicative interaction with maternal FAS was observed for rs2236222 and rs11849530 in relation to CHD risk. Additionally, the analysis of additive interaction between maternal FAS and offspring MTHFD1 and MTHFD2 genes was conducted. However, no additive interaction was found between any loci and maternal FAS (Table 6).

Furthermore, this study included a comprehensive stratified analysis to explore the influence of maternal FAS (Table 7). Among the maternal FAS group, rs2236222 (aOR: 1.48, 95CI%: 1.20, 1.84, *p* < 0.001), rs11849530(aOR: 0.72, 95CI%: 0.60, 0.87, *p* = 0.001), and rs828858 (aOR: 0.49, 95CI%: 0.36, 0.66, *p* < 0.001) exhibited statistical significance in relation to CHD risk. However, in the maternal group without FAS, only rs2236222 showed statistical significance (aOR: 3.74, 95CI%: 1.44, 9.71, *p* = 0.004), whereas rs11849530 and rs828858 did not (*p* > 0.05). 

## 4. Discussion

Since its introduction, perinatal FAS was recognized for its effectiveness and safety. While there are some theoretical considerations, such as the interaction between high FAS and vitamin B12, regarding anemia, cognition, and metabolism, no adverse effects have been identified with moderate FAS [27]. Pregnant women benefit from FAS as it helps prevent iron deficiency anemia, pre-eclampsia, gestational diabetes, and other complications [28,29,30,31]. Neural tube malformations in children are among the most common preventive effects of perinatal FAS [32,33]. Furthermore, FAS showed a protective effect against cardiovascular disease [34]. This study provided evidence supporting the association between maternal FAS and CHD and ASD. The adjusted odds ratios (aOR) for CHD and ASD were 0.51 (95%CI: 0.34–0.77) and 0.33 (95%CI: 0.19–0.58), respectively, indicating a potential reduction in disease risk with maternal FAS. These results were consistent with similar studies, suggesting that maternal FAS decreases the risk of CHD and its subtypes [35,36,37]. The mechanism behind this effect may involve potent antioxidant and antithrombotic properties, improvement of endothelial dysfunction [38], or prevention of low maternal folic acid levels, which can lead to homocysteine accumulation and interfere with normal cardiac neural crest development [39]. However, the study did not find any impact of maternal FAS on ventricular septal defect (VSD) and patent ductus arteriosus (PDA), which could be attributed to the limited sample size. It is noteworthy that FAS during the first and second trimesters increased the risk of CHD compared to supplementation three months prior to conception. Similar results were observed for ASD, VSD, and PDA. Prior research indicated that FAS before conception reduces the risk of spontaneous abortion, chromosomal abnormalities, and birth defects compared to supplementation after conception, with optimal maternal folic acid concentration [40,41,42]. However, in certain regions of China, women limited awareness and usage of folic acid, leading to a high prevalence of folic acid deficiency [15]. These findings underscored the importance of promoting folic acid awareness and appropriate usage, providing a new theoretical basis for guiding the timing of folic acid consumption in the future.

Several studies established associations between MTHFD1 and MTHFD2 gene variants and various health conditions such as cancer, cleft lip and palate, Down’s syndrome, and miscarriage [22,24,43,44,45]. This study focused on CHD and its subtypes as the outcomes of interest. The genetic variants rs2236222 and rs11849530 in the MTHFD1 gene, as well as rs828858 in the MTHFD2 gene, were found to be linked to CHD risk. Specifically, these three genetic loci were statistically significant for ASD and VSD, whereas no significant loci were observed in the PDA group. These results aligned with previous studies [46,47]. MacFarlane et al. demonstrated that inserting a gene trap vector into the MTHFD1 gene disrupted formyltetrahydrofolate synthase activity, leading to embryonic death in mice [48]. Beaudin et al. further corroborated these findings [49]. As for the MTHFD2 gene, Di Pietro et al. found that homozygous knockout mice experienced in utero death [50]. Studies investigating the association between polymorphisms in these two genes and CHD revealed a link between embryonic MTHFD1 gene and CHD incidence [21], but no relevant studies have explored the relationship between the MTHFD2 gene and CHD. Notably, the Arg653Gln variant protein in the MTHFD1 gene was found to have a shorter half-life than the wild-type protein, affecting cellular nucleotide metabolism. Population investigations further indicated that the Arg653Gln variant in MTHFD1 gene increased the risk of valvular defect aortic stenosis and conotruncal defects [51]. However, the results of some studies, including those by Shaw GM et al., Gong D et al., and Khatami M et al., did not support the association between rs2236222, rs11849530, rs828858, and CHD [19,25,52]. This study provides additional insights into the future understanding of CHD pathogenesis, and further validation in diverse populations is required to confirm these genetic loci as potential risk factors for offspring CHD.

This study also examined the interaction between folic acid intake and the rs828858 locus of the MTHFD2 gene in relation to the occurrence of CHD. Previous research demonstrated a strong synergistic effect between the MTHFD1 gene and maternal FAS on CHD [53]. Previous studies using hen models indicated that folic acid deficiency in hens could significantly increase MTHFD2 gene expression in their offspring, suggesting a potential correlation between maternal FAS and MTHFD2 gene expression in offspring [54]. However, there was limited research available to objectively determine the interaction between MTHFD2 gene and FAS in relation to CHD. The mechanisms proposed in this study were as follows. Firstly, MTHFD2 gene played a critical role in nucleic acid synthesis and stress response. FAS within this pathway may influence the progression of the stress response, thereby affecting the development of CHD. Additionally, FAS may impact methionine methylation, whereas the enzymes encoded by the MTHFD2 gene are involved in the methionine and folic acid cycles, suggesting a potential interaction between them. However, further investigation through animal experiments was required to elucidate the specific mechanisms involved.

This study had the advantage of thoroughly investigating the connection between MTHFD1 and MTHFD2 genes and CHD, including its subtypes, along with the interaction with maternal FAS. Additionally, attention was given to examining the association between the timing of FAS and the risk of CHD and its subtypes. The aim was to gain further insights into the pathogenesis of CHD by considering the interplay between genetic and environmental factors. However, there were certain limitations to acknowledge. Firstly, the study design adopted for this research was a hospital-based case–control study. Both the case and control groups were selected from different departments within the same hospital, potentially restricting the generalizability of the findings. Given the disparity in baseline characteristics between the case and control groups, adjustments were made to investigate the impact of maternal FAS, progeny MTHFD1, and MTHFD2 gene polymorphisms, along with their interactions, on congenital heart disease. Secondly, due to the limited sample size, it was not possible to explore the interaction between maternal FAS and MTHFD1 and MTHFD2 genes for each subtype, nor obtain information on the influence of paternal genetic factors on offspring CHD. Thirdly, concerning confounders of CHD, this study extensively addressed maternal environmental factors with established epidemiological foundations in existing literature. However, some other environmental factors influencing CHD, not encompassed in this study, could not be entirely ruled out. Fourthly, owing to limitations in the study design, the precise dosage and frequency of maternal exposure to perinatal pregnancy-related environmental factors could not be accurately recorded during the investigation. This aspect may potentially obscure the genuine impact of maternal exposure factors. Lastly, this study can only infer associations between environmental and genetic factors and cannot establish precise causal relationships. Future investigations should involve animal experiments and cohort studies to validate the connections between these factors and CHD.

## 5. Conclusions

In individuals of Chinese descent, our study identified associations between the risk of CHD and its subtypes, folic acid supplementation availability, and the timing of folic acid initiation. Additionally, we found significant associations between genetic variations at rs2236222, rs11849530, and rs828858 in the offspring MTHFD1 and MTHFD2 genes and CHD risk. Notably, a multiplicative interaction between rs828858 and maternal FAS was observed in relation to CHD risk. Stratified analyses further indicated that folic acid supplementation may mitigate the risk posed by genetic mutations. These findings suggest that pregnant women should carefully consider the timing of folic acid supplementation, and individuals with higher genetic risk may benefit from targeted folic acid supplementation as a preventive measure against CHD. However, the specific mechanisms underlying these associations remain unclear, warranting future extensive cohort studies in diverse populations to validate our study’s results.

## Figures and Tables

**Table 1:** Comparison of baseline characteristics between case and control groups.

Baseline Characteristics	Control Group (*n* = 620)	Case Group (*n* = 620)	*χ* ^2^	*p*
Residence				
Rural	342 (55.2%)	444 (71.6%)	36.153	<0.001
Urban	278 (44.8%)	176 (28.4%)		
Child sex				
Male	405 (65.6%)	303 (51.2%)	26.023	<0.001
Female	212 (34.4%)	289 (48.8%)		
Education level (years)				
<9	7 (1.1%)	87 (14.0%)	211.779	<0.001
9–12	117 (18.9%)	263 (42.4%)		
13–16	217 (35.0%)	167 (26.9%)		
≥17	279 (45.0%)	103 (16.6%)		
Pre-pregnancy BMI				
<18	156 (25.2%)	112 (18.1%)	11.758	0.003
18–24	356 (57.4%)	411 (66.3%)		
≥24	108 (17.4%)	97 (15.6%)		
History of stillbirth	2 (0.3%)	36 (5.8%)	31.383	<0.001
History of preterm birth	6 (1.0%)	17 (2.7%)	5.360	0.033
LBW history	5 (0.5%)	17 (2.7%)	9.961	0.002
Diabetes	25 (4.0%)	70 (11.3%)	23.084	<0.001
History of gestational diabetes	17 (2.7%)	63 (10.2%)	28.274	<0.001
History of gestational hypertension	9 (1.5%)	43 (6.9%)	23.204	<0.001
Antibiotic use during perinatal pregnancy	33 (5.3%)	92 (14.8%)	30.970	<0.001
Perinatal cold	132 (21.3%)	207 (33.4%)	22.836	<0.001
Perinatal fever	19 (3.1%)	59 (9.5%)	21.890	<0.001
Pregnancy smoking	6 (1.0%)	21 (3.4%)	8.519	0.005
Exposure to secondhand smoke during perinatal pregnancy	227 (36.6%)	327 (52.7%)	32.628	<0.001
Perinatal drinking	22 (3.5%)	62 (10.0%)	20.432	<0.001

LBW: low birth weight.

**Table 2:** Association of maternal folic acid intake for this pregnancy with CHD and its subtypes.

CHD and Its Subtypes	Crude-OR (95%CI)	Adjusted-OR (95%CI) *
Total CHD	0.42 (0.29, 0.61)	0.51 (0.34, 0.77)
ASD	0.27 (0.16, 0.45)	0.33 (0.19, 0.58)
VSD	0.67 (0.43, 1.03)	0.76 (0.47, 1.21)
PDA	0.55 (0.32, 0.97)	0.80 (0.42, 1.55)

CHD: congenital heart disease, ASD: atrial septal defect, VSD: ventricular septal defect, PDA: patent ductus arteriosus, CI: confidence interval, and OR: odds ratio. * Adjusted for residence, child sex, education level (years), pre-pregnancy BMI, history of stillbirth, history of preterm birth, LBW history, history of gestational diabetes, history of gestational hypertension, perinatal cold, perinatal fever, pregnancy smoking, exposure to secondhand smoke during perinatal pregnancy, and perinatal drinking.

**Table 3:** Association of time of starting to use folic acid for this pregnancy with the risk of CHD ant its subtype.

CHD and Its Subtypes	Crude-OR (95%CI)	Adjusted-OR (95%CI) *
Total CHD		
Three months prior to conception	1	1
First trimester of pregnancy	1.89 (1.42, 2.51)	1.80 (1.33, 2.43)
Second trimester of pregnancy	10.00 (3.74, 26.70)	10.43 (3.83, 28.35)
ASD		
Three months prior to conception	1	1
First trimester of pregnancy	4.65 (2.25, 9.65)	4.51 (2.18, 9.32)
Second trimester of pregnancy	8.04 (2.70, 23.89)	7.73 (2.59, 23.35)
VSD		
Three months prior to conception	1	1
First trimester of pregnancy	1.31 (0.97, 1.76)	1.16 (0.89, 1.58)
Second trimester of pregnancy	5.48 (2.50, 12.00)	5.36 (2.41, 11.88)
PDA		
Three months prior to conception	1	1
First trimester of pregnancy	1.92 (1.19, 3.10)	1.70 (1.05, 2.79)
Second trimester of pregnancy	3.44 (1.39, 8.55)	3.50 (1.39, 8.86)

CHD: congenital heart disease, ASD: atrial septal defect, VSD: ventricular septal defect, PDA: patent ductus arteriosus, CI: confidence interval, and OR: odds ratio. * Adjusted for residence, child sex, education level (years), pre-pregnancy BMI, history of stillbirth, history of preterm birth, LBW history, history of gestational diabetes, history of gestational hypertension, perinatal cold, perinatal fever, pregnancy smoking, exposure to secondhand smoke during perinatal pregnancy, and perinatal drinking.

**Table 4:** Association between offspring MTHFD1 and MTHFD2 genes variants and CHD and its subtypes.

SNPs	CHD (*n* = 620)			ASD (*n* = 139)			VSD (*n* = 448)			PDA (*n* = 168)		
	aOR (95%CI) *	*p*	Q_FDR_	aOR (95%CI) *	*p*	Q_FDR_	aOR (95%CI) *	*p*	Q_FDR_	aOR (95%CI) *	*p*	Q_FDR_
MTHFD1												
rs1950902												
GG	1			1			1			1		
GA	1.04 (0.80–1.35)	0.810	0.895	4.12 (0.91, 18.33)	0.063	0.189	0.92 (0.69, 1.21)	0.546	0.751	1.50 (1.00, 2.26)	0.051	0.396
AA	0.90 (0.60–1.34)	0.598	0.774	1.22 (0.60, 2.50)	0.579	0.643	0.76 (0.48, 1.19)	0.222	0.305	1.35 (0.70, 2.59)	0.367	0.573
Dominant model	1.06 (0.83, 1.35)	0.630	0.768	1.52 (1.00, 2.27)	0.052	0.262	0.85 (0.67, 1.08)	0.175	0.385	1.51 (1.08, 2.14)	0.020	0.143
Recessive model	0.98 (0.68, 1.40)	0.900	0.933	0.90 (0.51, 1.58)	0.702	0.780	0.79 (0.53, 1.16)	0.223	0.350	1.02 (0.62, 1.71)	0.929	0.929
Additive model	0.97 (0.80–1.17)	0.770	0.831	1.28 (0.94, 1.74)	0.101	0.183	0.89 (0.72, 1.08)	0.233	0.320	1.25 (0.93, 1.68)	0.118	0.597
rs2236225												
GG	1			1			1			1		
GA	1.02 (0.78–1.33)	0.855	0.895	1.10 (0.71, 1.68)	0.694	0.738	1.14 (0.86, 1.51)	0.371	0.583	1.32 (0.88, 1.94)	0.188	0.517
AA	0.85 (0.48–1.51)	0.586	0.774	1.83 (0.87, 3.87)	0.114	0.521	0.44 (0.21, 0.97)	0.043	0.118	1.09 (0.47, 2.53)	0.848	0.900
Dominant model	1.00 (0.78, 1.27)	0.983	0.983	1.35 (0.94, 1.94)	0.103	0.262	1.07 (0.83, 1.37)	0.614	0.675	1.52 (1.05, 2.20)	0.026	0.143
Recessive model	0.81 (0.47, 1.38)	0.414	0.745	1.97 (0.98, 3.95)	0.050	0.320	0.42 (0.20, 0.84)	0.015	0.083	1.23 (0.57, 2.58)	0.596	0.686
Additive model	0.98 (0.80–1.20)	0.831	0.831	1.23 (0.90, 1.69)	0.191	0.300	0.94 (0.74, 1.19)	0.589	0.648	1.17 (0.86, 1.60)	0.315	0.693
rs2236222												
AA	1			1			1			1		
GA	1.56 (1.21–2.02)	<0.001	<0.001	1.81 (1.20, 2.74)	0.007	0.030	1.57 (1.20, 2.08)	0.001	0.011	1.46 (0.97, 2.17)	0.072	0.396
GG	2.28 (1.27–4.04)	0.006	0.033	1.62 (0.57, 4.67)	0.361	0.521	2.19 (1.18, 4.04)	0.013	0.059	1.44 (0.56, 3.75)	0.451	0.584
Dominant model	1.70 (1.34, 2.18)	<0.001	<0.001	1.36 (0.95, 1.94)	0.092	0.262	1.51 (1.19, 1.93)	0.001	0.011	0.94 (0.67, 1.32)	0.720	0.880
Recessive model	1.94 (1.11, 3.38)	0.018	0.099	0.56 (0.21, 1.47)	0.246	0.492	1.75 (1.04, 2.94)	0.036	0.132	0.74 (0.35, 1.61)	0.462	0.686
Additive model	1.54 (1.26–1.89)	<0.001	<0.001	1.58 (1.12, 2.23)	0.009	0.033	1.52 (1.23, 1.92)	<0.001	<0.001	1.33 (0.96, 1.86)	0.079	0.597
rs11849530												
AA	1			1			1			1		
GA	0.89 (0.69–1.15)	0.371	0.583	0.76 (0.50, 1.15)	0.190	0.380	0.94 (0.71, 1.23)	0.638	0.780	1.03 (0.70, 1.54)	0.870	0.900
GG	0.46 (0.30–0.71)	<0.001	<0.001	0.36 (0.18, 0.77)	0.008	0.080	0.43 (0.26, 0.68)	<0.001	<0.001	0.53 (0.28, 1.03)	0.061	0.396
Dominant model	0.74 (0.60, 0.96)	0.020	0.073	0.78 (0.55, 1.11)	0.167	0.262	0.81 (0.63, 1.02)	0.073	0.341	1.02 (0.74, 1.42)	0.935	0.935
Recessive model	0.55 (0.37, 0.77)	0.001	0.011	0.57 (0.29, 1.12)	0.103	0.343	0.52 (0.34, 0.79)	0.002	0.022	0.73 (0.41, 1.29)	0.280	0.686
Additive model	0.75 (0.63–0.91)	0.002	0.007	0.67 (0.49, 0.89)	0.007	0.033	0.75 (0.61, 0.91)	0.004	0.022	0.83 (0.63, 1.08)	0.163	0.597
rs2236224												
GG	1			1			1			1		
GA	0.84 (0.65–1.08)	0.169	0.372	1.08 (0.70, 1.65)	0.739	0.739	0.97 (0.74, 1.29)	0.870	0.956	1.05 (0.71, 1.55)	0.818	0.900
AA	1.01 (0.65–1.54)	0.983	0.983	1.36 (0.71, 2.63)	0.355	0.521	0.94 (0.58, 1.52)	0.788	0.788	0.72 (0.35, 1.50)	0.383	0.573
Dominant model	0.88 (0.69, 1.11)	0.274	0.377	1.31 (0.91, 1.86)	0.150	0.262	1.10 (0.87, 1.40)	0.436	0.533	1.22 (0.87, 1.70)	0.269	0.592
Recessive model	1.02 (0.68, 1.52)	0.934	0.933	1.20 (0.67, 2.14)	0.540	0.771	0.82 (0.54, 1.24)	0.344	0.420	0.76 (0.41, 1.41)	0.385	0.686
Additive model	0.93 (0.78–1.20)	0.456	0.670	1.14 (0.85, 1.53)	0.397	0.485	0.97 (0.79, 1.19)	0.779	0.779	0.93 (0.69, 1.25)	0.628	0.768
rs1256142												
GG	1			1			1			1		
GA	1.28 (0.93–1.75)	0.130	0.358	0.90 (0.56, 1.44)	0.654	0.738	1.40 (0.98, 2.00)	0.061	0.224	2.08 (1.22, 3.53)	0.008	0.154
AA	1.53 (1.09–2.19)	0.015	0.066	0.72 (0.40, 1.27)	0.251	0.521	1.61 (1.08, 2.40)	0.016	0.059	1.57 (0.86, 2.86)	0.144	0.453
Dominant model	1.17 (0.89, 1.55)	0.261	0.377	0.74 (0.48, 1.11)	0.149	0.262	1.19 (0.90, 1.57)	0.216	0.396	1.56 (0.99, 2.44)	0.054	0.198
Recessive model	1.28 (0.99, 1.66)	0.063	0.173	0.65 (0.43, 1.02)	0.064	0.320	1.10 (0.84, 1.41)	0.548	0.603	0.79 (0.54, 1.16)	0.230	0.686
Additive model	1.23 (1.03–1.47)	0.016	0.041	0.85 (0.64, 1.13)	0.254	0.349	1.26 (1.04, 1.53)	0.019	0.052	1.17 (0.90, 1.55)	0.232	0.638
MTHFD2												
rs7571842												
GG	1			1			1			1		
GA	0.87 (0.67–1.12)	0.263	0.524	0.68 (0.44, 1.04)	0.077	0.141	0.86 (0.65, 1.13)	0.275	0.504	0.81 (0.54, 1.23)	0.321	0.573
AA	0.96 (0.64–1.43)	0.833	0.895	1.09 (0.58, 2.07)	0.784	0.784	0.73 (0.46, 1.15)	0.169	0.266	1.32 (0.73, 2.36)	0.375	0.573
Dominant model	0.84 (0.68, 1.09)	0.184	0.337	0.83 (0.58, 1.19)	0.324	0.356	0.81 (0.63, 1.04)	0.093	0.341	1.07 (0.77, 1.49)	0.694	0.880
Recessive model	0.92 (0.63, 1.34)	0.648	0.933	1.13 (0.64, 1.96)	0.696	0.780	0.66 (0.43, 1.00)	0.052	0.143	1.14 (0.68, 1.91)	0.626	0.686
Additive model	0.95 (0.79–1.12)	0.487	0.670	0.90 (0.67, 1.22)	0.513	0.564	0.86 (0.71, 1.04)	0.118	0.185	1.04 (0.78, 1.37)	0.816	0.900
rs702466												
CC	1			1			1			1		
GC	0.83 (0.65–1.08)	0.162	0.372	0.67 (0.43, 1.04)	0.071	0.141	0.83 (0.63, 1.11)	0.206	0.504	0.84 (0.56, 1.26)	0.391	0.573
GG	0.58 (0.29–1.12)	0.104	0.327	0.64 (0.21, 1.96)	0.437	0.546	0.48 (0.22, 1.05)	0.067	0.141	0.15 (0.69, 3.40)	0.296	0.573
Dominant model	0.84 (0.66, 1.07)	0.152	0.337	0.82 (0.57, 1.20)	0.312	0.356	0.91 (0.71, 1.16)	0.434	0.533	1.10 (0.78, 1.55)	0.583	0.880
Recessive model	0.63 (0.33, 1.22)	0.173	0.381	0.94 (0.33, 2.71)	0.913	0.913	0.59 (0.28, 1.23)	0.156	0.334	1.41 (0.61, 3.26)	0.424	0.686
Additive model	0.81 (0.65–1.00)	0.049	0.108	0.71 (0.49, 1.03)	0.073	0.183	0.78 (0.62, 1.00)	0.047	0.103	1.01 (0.74, 1.39)	0.950	0.950
rs828858												
TT	1			1			1			1		
TA	0.56 (0.41–0.76)	<0.001	<0.001	0.50 (0.29, 0.86)	0.012	0.044	0.67 (0.48, 0.92)	0.015	0.083	0.66 (0.40, 1.07)	0.092	0.405
AA	0.59 (0.19–1.81)	0.355	0.583	-	-		0.54 (0.14, 2.04)	0.360	0.440	2.40 (0.77, 7.49)	0.133	0.453
Dominant model	0.59 (0.44, 0.79)	<0.001	<0.001	0.66 (0.41, 1.07)	0.091	0.262	0.81 (0.60, 1.09)	0.158	0.385	0.97 (0.64, 1.45)	0.863	0.935
Recessive model	0.67 (0.24, 2.03)	0.474	0.745	-	-		0.88 (0.69, 1.14)	0.334	0.420	3.16 (1.02, 9.77)	0.046	0.396
Additive model	0.61 (0.45–0.79)	<0.001	<0.001	0.47 (0.28, 0.78)	0.004	0.033	0.68 (0.50, 0.92)	0.011	0.040	0.89 (0.59, 1.32)	0.551	0.758
rs828903												
AA	1			1			1			1		
AG	0.88 (0.68–1.14)	0.339	0.583	0.77 (0.49, 1.20)	0.243	0.334	0.85 (0.63, 1.13)	0.254	0.504	0.97 (0.64, 1.47)	0.900	0.900
GG	0.57 (0.32–1.02)	0.061	0.220	0.47 (0.16, 1.39)	0.170	0.521	0.56 (0.29, 1.07)	0.077	0.141	1.48 (0.72, 3.05)	0.284	0.573
Dominant model	0.85 (0.66, 1.08)	0.178	0.337	0.87 (0.60, 1.27)	0.468	0.468	0.88 (0.69, 1.14)	0.334	0.525	1.24 (0.88, 1.75)	0.213	0.586
Recessive model	0.58 (0.33, 1.03)	0.061	0.173	0.62 (0.22, 1.74)	0.361	0.602	0.66 (0.36, 1.22)	0.182	0.334	1.87 (0.95, 3.68)	0.072	0.396
Additive model	0.82 (0.67–1.01)	0.065	0.119	0.73 (0.51, 1.05)	0.091	0.183	0.80 (0.64, 1.01)	0.056	0.103	1.11 (0.82, 1.49)	0.514	0.758
rs1095966												
CC	1			1			1			1		
CA	0.96 (0.74–1.26)	0.827	0.895	0.68 (0.44, 1.04)	0.075	0.141	1.02 (0.75, 1.35)	0.956	0.956	1.04 (0.69, 1.59)	0.830	0.900
AA	1.10 (0.78–1.55)	0.579	0.774	1.28 (0.75, 2.20)	0.365	0.521	1.17 (0.80, 1.71)	0.424	0.466	1.24 (0.72, 2.15)	0.434	0.584
Dominant model	1.03 (0.81, 1.32)	0.755	0.832	0.80 (0.56, 1.15)	0.233	0.320	1.06 (0.82, 1.34)	0.699	0.699	1.11 (0.78, 1.57)	0.580	0.880
Recessive model	1.04 (0.76, 1.41)	0.812	0.891	1.32 (0.86, 2.05)	0.226	0.492	1.04 (0.76, 1.42)	0.813	0.813	0.86 (0.55, 1.36)	0.522	0.686
Additive model	1.04 (0.88–1.22)	0.680	0.831	1.04 (0.78, 1.37)	0.804	0.804	1.08 (0.89, 1.29)	0.486	0.594	1.10 (0.85, 1.46)	0.472	0.758

CHD: congenital heart disease, ASD: atrial septal defect, VSD: ventricular septal defect, PDA: patent ductus arteriosus, CI: confidence interval, OR: odds ratio, and Q_FDR_: false discovery rate *p* value. * Adjusted for residence, child sex, education level (years), pre-pregnancy BMI, history of stillbirth, history of preterm birth, LBW history, history of gestational diabetes, history of gestational hypertension, perinatal cold, perinatal fever, pregnancy smoking, exposure to secondhand smoke during perinatal pregnancy, and perinatal drinking.

**Table 5:** Multiplicative interactions between the offspring MTHFD1 and MTHFD2 genes and maternal folic acid use on the risk of total CHD.

	Crude-OR (95%CI)	*p*	Adjusted-OR (95%CI) *	*p*
rs2236222	0.65 (0.37, 1.13)	0.126	0.78 (0.43, 1.35)	0.343
rs11849530	0.70 (0.39, 1.26)	0.238	0.67 (0.37, 1.22)	0.187
rs828858	0.29 (0.12, 0.72)	0.007	0.26 (0.10, 0.65)	0.004

CHD: congenital heart disease, CI: confidence interval, and OR: odds ratio. * Adjusted for residence, child sex, education level (years), pre-pregnancy BMI, history of stillbirth, history of preterm birth, LBW history, history of gestational diabetes, history of gestational hypertension, perinatal cold, perinatal fever, pregnancy smoking, exposure to secondhand smoke during perinatal pregnancy, and perinatal drinking.

**Table 6:** Additive interactions between the offspring MTHFD1 and MTHFD2 genes and maternal folic acid use on the risk of total CHD.

	RERI	AP
rs2236222	−3.00 (−7.63, 1.63)	−6.81 (−23.04, 9.42)
rs2236222 *	−2.47 (−6.65, 1.79)	−4.75 (−16.86, 7.45)
rs11849530	−0.02 (−1.83, 1.79)	−0.03 (−2.62, 2.55)
rs11849530 *	0.17 (−1.63, 1.94)	0.22 (−1.94, 2.39)
rs828858	−2.47 (−6.76, 1.83)	−8.39 (−30.43, 13.64)
rs828858*	−2.95 (−7.74, 1.88)	−11.17 (−39.24, 16.87)

CHD: congenital heart disease, RERI: relative excess risk due to interaction, and AP: attributable proportion. * Adjusted for residence, child sex, education level (years), pre-pregnancy BMI, history of stillbirth, history of preterm birth, LBW history, history of gestational diabetes, history of gestational hypertension, perinatal cold, perinatal fever, pregnancy smoking, exposure to secondhand smoke during perinatal pregnancy, and perinatal drinking.

**Table 7:** Maternal MTHFD genotype by stratification of maternal folic acid use and risk of CHD.

SNPs	Crude-OR (95%CI)	*p*	Adjusted-OR (95%CI) *	*p*
FAS (*n* = 1103)				
rs2236222	1.49 (1.17, 1.89)	<0.001	1.48 (1.20, 1.84)	<0.001
rs11849530	0.65 (0.51, 0.83)	<0.001	0.72 (0.60, 0.87)	0.001
rs828858	0.51 (0.37, 0.69)	<0.001	0.49 (0.36, 0.66)	<0.001
No FAS (*n* = 137)				
rs2236222	3.38 (1.42, 8.08)	0.006	3.74 (1.44, 9.71)	0.004
rs11849530	1.34 (0.64, 2.78)	0.440	1.58 (0.71, 3.50)	0.264
rs828858	1.86 (0.79, 4.36)	0.153	1.55 (0.50, 4.77)	0.448

CHD: congenital heart disease, FAS: folic acid supplementation, CI: confidence interval, and OR: odds ratio. * Adjusted for residence, child sex, education level (years), pre-pregnancy BMI, history of stillbirth, history of preterm birth, LBW history, history of gestational diabetes, history of gestational hypertension, perinatal cold, perinatal fever, pregnancy smoking, exposure to secondhand smoke during perinatal pregnancy, and perinatal drinking.

## Data Availability

Data is unavailable due to privacy or ethical restrictions.

## Supplementary Materials

The following supporting information can be downloaded at: https://www.mdpi.com/article/10.3390/nu15163502/s1, Figure S1: The flowchart of this study; Table S1: Sample size estimation of MTHFD1 and MTHFD2 gene representative loci; Table S2: Basic information of the candidate genetic loci for MTHFD gene; Table S3: Maternal folate use information for this pregnancy across groups; Table S4: Distribution frequency of MTHFD genotype and Hardy-Weinberg balance test.
