# Supplementary material for: Association of Maternal Folate Intake and Offspring MTHFD1 and MTHFD2 Genes with Congenital Heart Disease
Source: Nutrients. 2023 Aug 9;15(16):3502. doi: 10.3390/nu15163502 (PMC10458540; doi:10.3390/nu15163502)
Supplement: Supplementary file 1 [file nutrients-15-03502-s001.zip › nutrients-2506639-supplementary.pdf]

Table S1. Sample size estimation of MTHFD1 and MTHFD2 gene representative loci

Gene	Representative loci	Mutation rate (%)		Minimum sample size required for case/control group
		Case group	Control group	
MTHFD1				
	rs2236225*	65	50	271
	rs2236224 and rs1256142**	29.4	43.7	281
MTHFD2				
	rs828858 and rs1667627**	44.2	33.6	530

*Based on reference 22.

** Based on reference 23.

Table S2. Basic information of the candidate genetic loci for MTHFD gene

Gene	Representative loci	Chromosome (GRCh38)	Major allele	Minor allele	MAF	
					Global*	East Asian**
MTHFD1	rs1950902	14:64415662	G	A	0.177	0.355
	rs2236225	14:64442127	G	A	0.342	0.198
	rs2236222	14:64448464	A	G	0.086	0.220
	rs11849530	14:64451694	A	G	0.214	0.360
	rs1256146	14:64453947	G	A	0.171	0.110
	rs2236224	14:64442433	G	A	0.308	0.291
	rs1256142	14:64444076	G	A	0.426	0.460
	rs34616731	14:64462569	T	A	0.179	0.124
MTHFD2	rs7571842	2:74233777	G	A	0.435	0.344
	rs702466	2:74142823	C	G	0.239	0.245
	rs828858	2:74195067	T	A	0.217	0.146
	rs828903	2:74209594	A	G	0.420	0.255
	rs1095966	2:74192260	C	A	0.384	0.400

MAF minor allele frequency

*Based on the total population MAF provided by the 1000Genomes project;

**Based on East Asian population MAF provided by the 1000Genomes project.

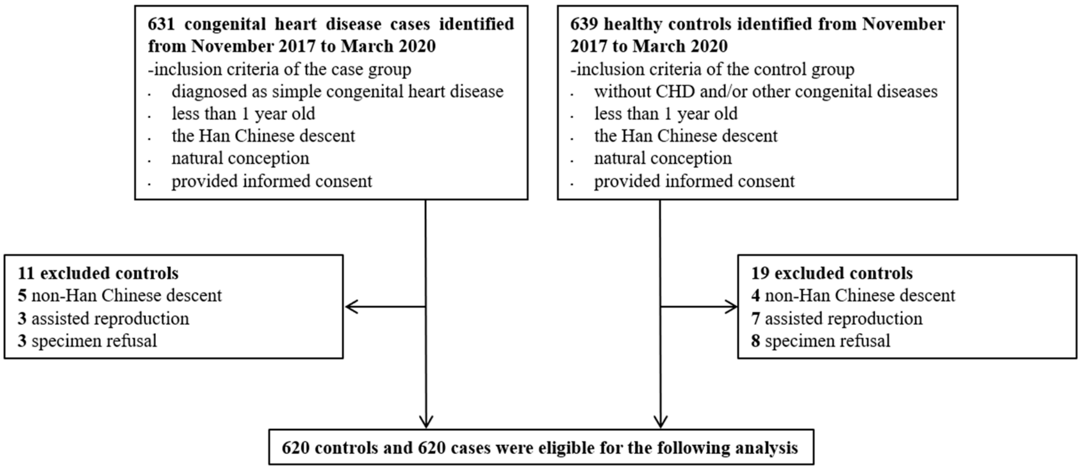

Figure S1. The flowchart of this study

Table S3. Maternal folate use information for this pregnancy across groups

Groups	Use of folic acid for this pregnancy		Time of starting to use folic acid for this pregnancy		
	yes	no	Three months prior to conception	First trimester of pregnancy	Second trimester of pregnancy
Control	577(93.1%)	43(6.9%)	162(28.1%)	410(71.0%)	5(0.9%)
Total CHD	526(84.8%)	94(15.2%)	94(17.9%)	403(76.6%)	29(5.5%)
ASD	109(78.4%)	30(21.6%)	8(7.3%)	94(86.2%)	7(6.4%)
VSD	403(90.0%)	45(10.0%)	78(19.4%)	301(74.7%)	24(6.0%)
PDA	148(88.1%)	20(11.9%)	21(14.2%)	119(80.4%)	8(5.4%)

CHD congenital heart disease, ASD atrial septal defect, VSD ventricular septal defect, PDA patent ductus arteriosus

Table S4. Distribution frequency of MTHFD genotype and Hardy-Weinberg balance test

SNPs		Case group (n=620)	Control group (n=620)	χ^2	P	Q_{FDR}
MTHFD1						
	GG	259(41.8%)	251(40.5%)	0.113	0.737	0.803
rs1950902	GA	290(46.8%)	290(46.8%)			
	AA	71(11.5%)	79(12.7%)			
	GG	393(63.4%)	403(65.0%)	3.068	0.080	0.327
rs2236225	GA	201(32.4%)	185(29.8%)			
	AA	26(4.2%)	32(5.2%)			
	AA	314(50.6%)	378(61.0%)	2.139	0.143	0.327
rs2236222	GA	262(42.3%)	220(35.5%)			
	GG	44(7.1%)	22(3.5%)			
	AA	304(49.0%)	253(40.8%)	0.997	0.318	0.503
rs11849530	GA	264(42.6%)	277(44.7%)			
	GG	52(8.4%)	90(14.5%)			
	GG	490(79.0%)	469(75.6%)	7.629	0.006	0.039
rs1256146	GA	127(20.5%)	149(24.0%)			
	AA	3(0.5%)	2(0.3%)			
	GG	330(53.2%)	329(53.1%)	1.443	0.230	0.427
rs2236224	GA	230(37.1%)	237(38.2%)			
	AA	60(9.7%)	54(8.7%)			
	GG	119(19.2%)	135(21.8%)	0.109	0.741	0.803
rs1256142	GA	315(50.8%)	313(50.5%)			
	AA	186(30.0%)	172(27.7%)			
rs34616731	TT	465(75.0%)	461(74.4%)	9.027	0.003	0.039

	AT	147(23.7%)	157(25.3%)			
	AA	8(1.3%)	2(0.3%)			
MTHFD2						
	GG	307(49.5%)	274(44.2%)	0.040	0.841	0.841
rs7571842	GA	249(40.2%)	278(44.8%)			
	AA	64(10.3%)	68(11.0%)			
	CC	399(64.4%)	377(60.8%)	0.882	0.348	0.503
rs702466	GC	205(33.1%)	218(35.2%)			
	GG	16(2.6%)	25(4.0%)			
	TT	512(82.6%)	465(75.0%)	0.417	0.518	0.673
rs828858	TA	103(16.6%)	146(23.5%)			
	AA	5(0.8%)	9(1.5%)			
	AA	405(65.3%)	388(62.6%)	2.210	0.137	0.327
rs828903	AG	194(31.5%)	197(31.8%)			
	GG	21(3.4%)	35(5.6%)			
	CC	232(37.4%)	230(37.1%)	2.061	0.151	0.327
rs1095966	CA	281(45.3%)	281(45.3%)			
	AA	107(17.3%)	109(17.6%)			

SNP single nucleotide polymorphism, Q_{FDR} false discovery rate P value
